# Liquid Phase Assisted Superplastic Deformation of TiO_2_-Doped ZTA Ceramics

**DOI:** 10.3390/ma12132050

**Published:** 2019-06-26

**Authors:** Yufei Zu, Guoqing Chen, Xuesong Fu, Wenlong Zhou

**Affiliations:** 1 Key Laboratory of Advanced Technology for Aerospace Vehicles of Liaoning Province, School of Aeronautics and Astronautics, Dalian University of Technology, Dalian 116085, China; 2 Key Laboratory of Solidification Control and Digital Preparation Technology (Liaoning Province), School of Materials Science and Engineering, Dalian University of Technology, Dalian 116085, China

**Keywords:** ZrO_2_ toughened Al_2_O_3_ ceramic, grain boundary feature, superplastic deformation, TiO_2_ dopants

## Abstract

In this study, the compressive deformation of zirconia toughened alumina (ZTA) ceramics doped with different amounts of TiO_2_ dopants were investigated in the temperature range of 1300–1400 °C to evaluate the stress exponent (*n* value) and apparent deformation activation energy (*Q* value). With 0–8 wt.% TiO_2_ dopants, the *n* values and *Q* values of the TiO_2_-doped ZTA ceramics were calculated as 2–3 and 605–749 kJ/mol, respectively. Moreover, three grain boundary features were observed in these deformed materials, named the clean grain boundary, thin liquid phase grain boundary, and thick liquid phase grain boundary. Based on the deformation behavior and microstructure evolution, it was found that the lower apparent activation energy and higher strain rate of TiO_2_-doped ZTA ceramics are intensively related to the grain boundary feature.

## 1. Introduction

Oxide ceramics are typical structural materials with wide applications, mainly used in cutting tools, bearings, high temperature engine components, and knee replacement prostheses, owing to their excellent ambient and high temperature mechanical properties. However, due to their strong chemical bonding and low diffusion coefficient, it is difficult to fabricate the sophisticated parts through forming methods like metals [1,2,3]. In 1986, Wakai and co-workers [4] reported the superplasticity of polycrystalline ceramics for the first time. Since then, some fine-grain ceramics have been regarded as superplastic materials at elevated temperature. Thus, in recent years, superplastic formation (SPF; including forging, extrusion, bending, stretching, and gas bulging forming) has been an appropriate technology to manufacture ceramic parts with complex shapes.

ZrO_2_ toughened Al_2_O_3_ ceramic (ZTA) is an outstanding structural ceramic due to its excellent mechanical properties [5,6] and good superplastic deformability. Superplasitc deformation and net-shape forming of ZTA ceramics have been investigated by many researchers [7,8,9,10,11,12]. Wakai et al. [12] investigated the superplastic deformation of ZTA ceramic, and found that its limited elongation is about 200% during deformation at 1450 °C. Chen and co-workers [10] reported that the dense ZTA samples can be deep-drawn to a dome height of at least 12 mm at a high punch rate of 0.6 mm/min at 1400 °C. In most oxide ceramics, the superplastic deformation always needs a lower strain rate of <10^−5^ s^−1^ and a higher deformation temperature of >1450 °C. Obviously, for industrial applications, the superplastic formation needs a higher efficiency (higher strain rate) and a lower production cost (lower deformation temperature) [13]. This means that the superplasticity of oxide ceramics should be enhanced to accommodate the industrial demands.

To improve the superplasticity of oxide ceramics, attempts have made been using dopants [14]. Doping TiO_2_ and MgO into ZrO_2_ can achieve a large strain of 206% at a higher strain rate of 1.2 × 10^−4^ s^−1^ and a relatively lower temperature of 1350 °C [15]. Ti/Mn co-doped Al_2_O_3_ with 3 mol. % ZrO_2_ can be shape-formed to 100% strain at a temperature as low as 1280 °C [16]. In our previous studies [17], it was found out that introducing 8 wt.% TiO_2_ into the ZTA ceramics can increase the strain rate by two orders of magnitude. The dominant deformation mechanism of the TiO_2_-doped ZTA is grain boundary sliding. In fact, for the deformation of the polycrystalline ceramics, when the grains slide over each other, stress concentration will occur at grain boundaries and triple junctions [14]. Hence, other sufficient accommodation (e.g., diffusion, dislocation migration) is needed to inhibit cavity nucleation induced by stress concentration. During the high temperature superplastic deformation, diffusion is a momentous accommodation mechanism that relaxes the stress concentration. Generally, the deformed apparent activation energy can be used for evaluating the accommodated diffusion process [18,19]. Sakka [15] calculated the apparent deformation activation energy in Ti/Mn-doped ZrO_2_ ceramic as 411 kJ/mol, which is close to the grain growth activation energy (447 kJ/mol). Combined with the microstructure, they found that Ti and Mn dopants can improve the cations’ diffusion, grain growth, and dislocation recovery during deformation. Especially when liquid phase occurs along the grain boundaries due to doping, the diffusion process during deformation may be changed. In the Al_2_O_3_ and ZTA ceramics [20,21,22] with (CuO-TiO_2_-B2O_3_-MgO) liquid phase, the grain boundary diffusion was enhanced significantly, which is in accordance with obvious alteration of the apparent deformation activation energy. Therefore, for the TiO_2_-doped ZTA ceramics with outstanding advantages, further investigation of the relationship between the apparent deformation activation energy and grain boundary feature evolution during deformation is crucial to discuss the deformation accommodation mechanisms.

In the current study, the compressive deformation of the TiO_2_-doped ZTA ceramics at different temperatures of 1300–1400 °C were conducted to estimate their apparent deformation activation energy for determination of the respective diffusion path. Moreover, the grain boundary features and their evolution during the superplastic deformation were also investigated to clarify the accommodation deformation mechanisms.

## 2. Materials and Methods

The raw materials Al_2_O_3_ and ZrO_2_ were synthesized at a mass ratio of 58:42 by heating ethanol-aqueous salt solutions [23,24]. The particle size of Al_2_O_3_ and ZrO_2_ powders is approximately 50 nm. Commercial rutile TiO_2_ (Nanjing Guanye Co. Ltd., Nanjing, China) purity ≥99.8%, particle size ≤ 80 nm) was selected as the dopant in this study (the doping contents are 0, 1, 4, and 8 wt.%, denoted as 0T, 1T, 4T, and 8T, respectively). Then, the mixed powders were sintered at 1400 °C to be used for investigation of compressive deformation. The grain size and grain shape were dependent on the TiO_2_ content, as the reference reported [17]. The detail methodology of sintering TiO_2_-doped ZTA can be found in our previous study [25]. To evaluate the stress exponent and apparent activation energy, the compression deformation was conducted at 1300 °C and 1350 °C by using the “stress-jump” method. The deformation behavior of TiO_2_-doped ZTA ceramics at 1400 °C was used as reference [17]. The deformation mold was made of high-strength graphite. When the deformation temperature reached the given value, the specimens were kept at the set temperature for 10 min before compressive testing. The temperature during deformation was measured using a platinum-rhodium thermocouple. The deformation test was conducted by using high temperature vacuum sintering furnaces. The deformation parameters in superplastic ceramics are calculated by the following constitutive equation:

(1) $$\dot{\varepsilon }=A\cdot \frac{\sigma^{n}}{d^{p}}\cdot \exp\left(-\frac{Q}{RT}\right)$$

where $\dot{\varepsilon }$ is the strain rate, *A* is a constant, *σ* is the flow stress, *n* is the stress exponent, *d* is the grain size, *p* is the grain size exponent, *Q* is the activation energy, *R* is the gas constant, and *T* is the absolute temperature. The compressive strain *ε*, the flow stress *σ*, and the strain rate $\dot{\varepsilon }$ were calculated by the directly measured load *P*, immediate *H*_x_, initial height *H*_0_, and initial end face area *A*_0_, as a previous study [17] reported.

The microstructure was characterized by means of scanning electron microscopy (SEM, Supra-55, Carl Zeiss Sigma NTS Gmbh, Jena, Germany). The SEM specimens were polished with a diamond paste of 1.5 μm and then thermally etched in air for 1 h at a 50 °C lower temperature than the deformation temperature of each specimen. The average grain sizes of the two phases were calculated using the linear intercept method: *d* = 1.56*L*, *L* is the intercept length. The grain boundary feature was characterized by using transmission electron microscope (TEM, Tecnai G220, FEI, Eindhoven, the Netherlands). The TEM thin foils were cut for *Φ* 3 mm disks from the different TiO_2_-doped ZTA ceramics. The foils were pre-thinned down to a few micrometers thickness by dimpling. A final thinning was performed by gentle ion milling.

## 3. Results and Discussion

**Figure 1:**
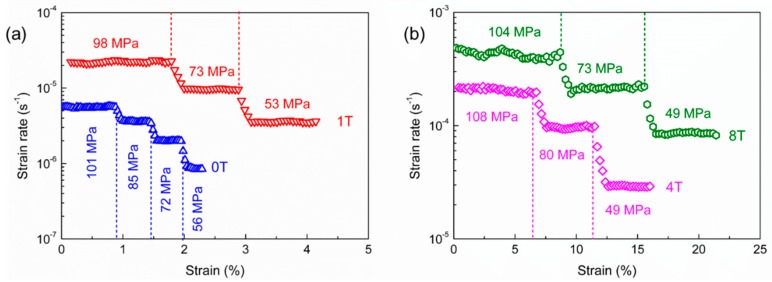
Curves of strain rate with strain at different flow stress for the four TiO_2_-doped ZTA ceramics conducted under 1300 °C: (**a**) 0T and 1T, (**b**) 4T and 8T.

**Figure 2:**
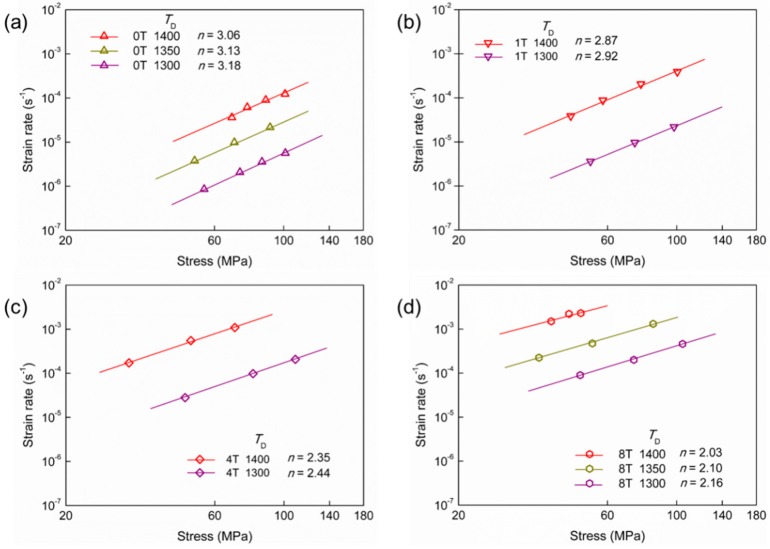
Stress-strain rate relationship of (**a**) 0T, (**b**) 1T, (**c**) 4T and (**d**) 8T under the deformation at 1300–1400 °C.

The compressive deformations of the TiO_2_-doped ZTA ceramics (0T, 1T, 4T, and 8T) were conducted in this study. To investigate the deformation behavior at different flow stress levels, the applied force was changed two or three times in one deformation testing at a given deformation temperature. Figure 1 shows the strain rate with strain at different flow stress levels for the four TiO_2_-doped ZTA ceramics conducted under 1300 °C. As can be seen in Figure 1, when the applied force was just changed, the transition deformation occurred first. A moment later, a relatively steady-state deformation occurred subsequently, as shown in Figure 1. In this study, the applied force was not changed until the occurrence of a short steady-state deformation. Considering that the cross sectional area of the specimens was gradually changed with deformation strain, the flow stress *σ*_flow_ was approximately modified according to the following equation:

(2) σflow=Fapplied·hxS0·h0.

where *h*_x_ is the height of the specimen at a certain moment, *S*_0_ and *h*_0_ are the initial area of the end face and the initial height of the specimen, respectively. All flow stresses reported in this paper have been already modified by using the upper equation. It is shown that the measured strain rate was obviously related to the applied stress. For the comparative analysis of the deformation behaviors of 0T–8T at different temperatures, the relationships between flow stress and strain rate are plotted on a logarithmic scale in Figure 2. In this plot, the flow stress and strain rate are only taken from the steady-state deformation stages. As can be seen in Figure 2, with the decrease of the deformation temperature, the strain rate at the same flow stress decreased. In the previous studies, during the superplastic deformation at the higher temperature of 1400 °C, the grain growth is extremely slight. Thus, the influence of the grain growth during the deformation is not considered in the present study. In a word, the grain size *d*, grain growth exponent *p*, and the activation energy *Q* in the same specimen are assumed as constants. Therefore, the stress exponent (*n* value) is calculated from the slope lines according to $\dot{\varepsilon }=A\cdot \sigma^{n}$. In the present study, it is found that the *n* values of the same material are almost equal at each deformed temperature. The values of stress exponents are in the range of 3.06–3.18 for 0T, 2.87–2.92 for 1T, 2.35–2.44 for 4T, and 2.03–2.16 for 8T, respectively. It is indicated that the deformation mechanism of the same material may be unchanged in the range of 1300–1400 °C.

**Figure 3:**
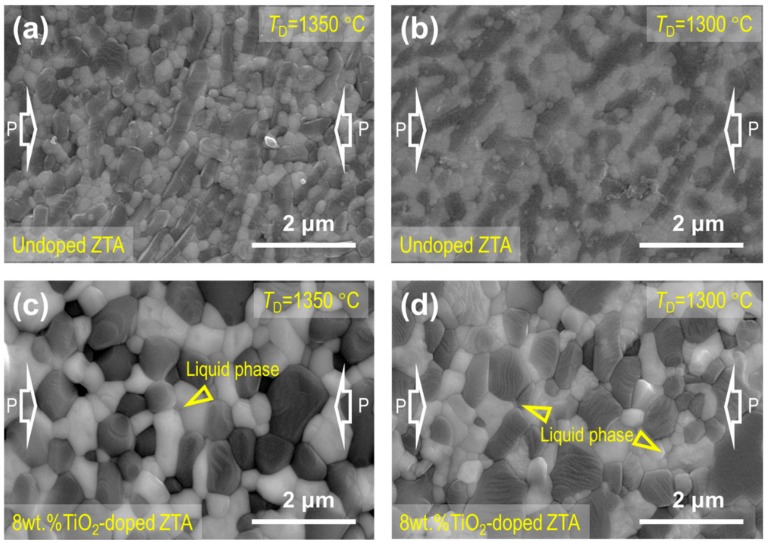
Microstructures of the (**a**–**b**) undoped and (**c**–**d**) 8 wt.%TiO_2_-doped ceramics after deformation. The compression stress axis is marked by the white hollow arrows.

**Figure 4:**
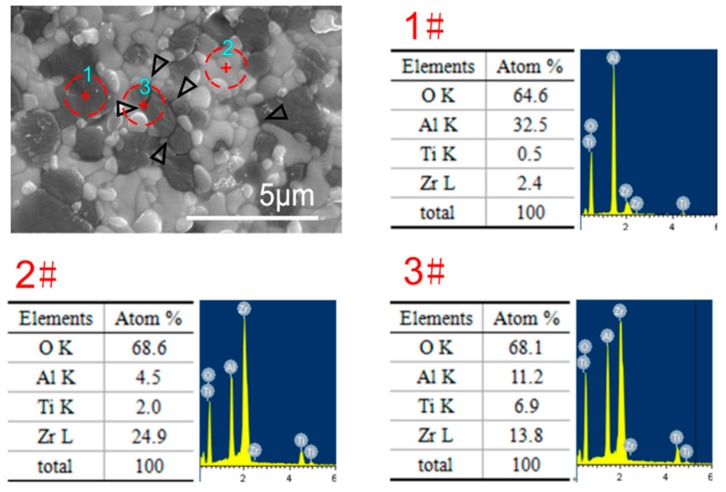
Microstructure and EDS analysis (1# is Al_2_O_3_, 2# is ZrO_2_, and 3# is liquid phase).

The typical microstructures of the undoped and 8 wt.% TiO_2_-doped ZTA ceramics after deformation at 1300 °C and 1350 °C are shown in Figure 3. Compared with the undeformed [25] and 1400 °C deformed specimens [17], the average grain size and grain shape are almost unchanged for each material. For the undoped ZTA ceramic, no conspicuous texture of the elongated Al_2_O_3_ grains was formed, such as the similar material deformed at 1400 °C in the previous study [17]. This may be because at the lower deformed temperatures of 1300 °C and 1350 °C, the strain rate of ZTA is extremely low, and the accumulated strain in the whole deformation is too small to form the conspicuous texture of the elongated Al_2_O_3_ grains. For the 8 wt.% TiO_2_-doped ZTA ceramics, whether deformed at 1300 °C, 1350 °C, or 1400 °C, there is no obvious difference in grain size, grain shape, or grain boundary liquid phase. To understand the element composition of the liquid phase, the SEM-EDS (Energy Dispersive Spectrometer) results are shown in in Figure 4. However, because the diameter of the region detected by SEM-EDS is larger than the liquid phase, it is difficult to examine the composition of the liquid phase accurately. Nevertheless, the SEM-EDS results can still be used for contrasting analysis of the element composition in different regions. As can be seen in Figure 4, the regions of 1#, 2#, and 3# are located in the Al_2_O_3_ grain, ZrO_2_ grain, and liquid phase, respectively. The Ti concentrations in Al_2_O_3_ and ZrO_2_ are detected as 0.5 mol.% and 2 mol.%, which are basically in agreement with the limited solubility of TiO_2_ in Al_2_O_3_ (<0.27 wt.%) and ZrO_2_ (<16 wt.%). The higher Ti concentration of 6.9 mol.% in the liquid phase indicates that the liquid phase is rich in Ti element. In particular, the real value of Ti concentration in the liquid phase should be much higher than the measured value (6.9 mol.%), because the region 3# includes the unsolicited regions located at Al_2_O_3_ and ZrO_2_ grains. Many Ti cations segregated on the grain boundary may be the reason of the formation of grain boundary liquid phase. Notably, in the previous studies, the lowest temperature of the Al_2_O_3_-ZrO_2_-TiO_2_ system is 1580–1610 °C, which is higher than the sintering and deformation temperature of 1300–1400 °C. Thus, the grain boundary liquid phases in this study are more likely to be generated owing to the solid-state phase transformation below the bulk eutectic temperature. Combined with the similar *n* values for each individual material, the deformation mechanism is not altered with the temperature changing at 1300–1400 °C. Based on the microstructure evolution and *n* values in the range of 2–3, the dominant deformation mechanism is grain boundary sliding. For the polycrystalline ceramics, when the grains slide over each other, stress concentration will occur at grain boundaries and triple junctions. If the strain rate is unduly fast, some cavities will nucleate and then form cracks at grain boundaries during superplastic deformation. Hence, grain boundary sliding must be accommodated by another process in order to keep the grains from separating. It has been proposed that rapid and sufficient dislocation motion or diffusion is necessary during deformation to prevent cavity formation [14,26]. Generally, the deformed apparent activation energy can be used to evaluate the accommodated process. Therefore, the alterations of the deformed apparent activation energy and grain boundary feature are discussed as follows.

**Figure 5:**
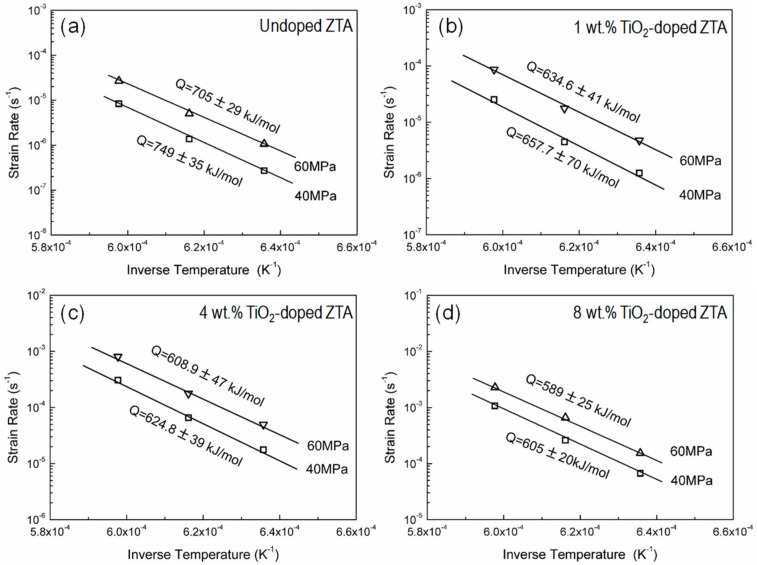
Relationships between the strain rate and reciprocal absolute temperature for the ZTA ceramics doped with different amounts of TiO_2_: (**a**) 0T, (**b**) 1T, (**c**) 4T and (**d**) 8T.

Figure 5 shows the relationship between the strain rate and reciprocal absolute temperature for the ZTA ceramics doped with different amount of TiO_2_. The slope of the straight line obtained from an Arrhenius type is used to determine the apparent deformation activation energy of the high temperature deformation. The values of the apparent activation energy in the undoped ZTA ceramic are calculated as 749 ± 35 kJ/mol (under 40 MPa) and 705 ± 29 kJ/mol (under 60 MPa), which are higher than those of pure Al_2_O_3_ (420–490 kJ/mol) [27] and pure ZrO_2_ (500–600 kJ/mol) [28] ceramics. The higher *Q* value in ZTA is probably related to the diffusion of species along the ZrO_2_-Al_2_O_3_ interface (inter-diffusion phenomena between Al_2_O_3_ and ZrO_2_ grains) [29]. The sliding mobility of Al_2_O_3_-Al_2_O_3_ interfaces may be reduced by the segregation of Zr^4+^ along these interfaces [30]. The apparent deformation activation energy (705–749 kJ/mol) in the undoped ZTA ceramic is very close to the activation energy of sintering (700 ± 100 kJ/mol) [31], superplasticity (740 kJ/mol) [32], and grain growth (732 kJ/mol) [32] in the typical ZTA ceramics, which are in agreement with those for grain boundary diffusion controlled by interface reaction reported in the ZTA ceramics with clean grain boundaries [32].

**Figure 6:**
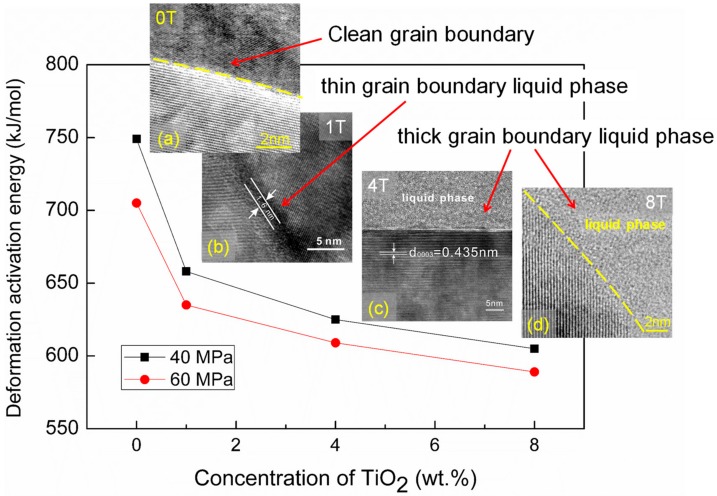
The relationship between deformed apparent activation energy and grain boundary feature in the four TiO_2_-doped ZTA ceramics. Grain boundary features were determined by high resultion TEM (HR-TEM) micrographs at typical grain boundaries. Three grain boundary features were observed, named the clean grain boundary (**a**) in 0T, thin liquid phase (**b**) in 1T, and thick liquid phase (**c**–**d**) in 4T and 8T.

For the ZTA doped with 1–8 wt.% TiO_2_, the apparent activation energies (Q values) are calculated as *Q*_1T_ = 635–658 kJ/mol, *Q*_4T_ = 609–625 kJ/mol, and *Q*_8T_ = 589–605 kJ/mol, respectively. With the increase of TiO_2_ concentration, the apparent activation energy of the TiO_2_-doped ZTA ceramics decreased, which indicates that the interfacial diffusion during deformation may be changed by TiO_2_ doping. Considering the occurrence of the glassy phase in the TiO_2_-doped ZTA [25], the alteration of the activation energy may be influenced by the grain boundary feature dependent on TiO_2_ content. Generally, the alteration of grain boundary feature may affect the grain boundary transport rate. Harmer and his co-workers [33,34] summarized the grain boundary migration with different features for Al_2_O_3_ ceramics doped with various dopants. Their results indicated that the mass transport rate on the grain boundary increases with an increase of the width of the grain boundary phase [33,34]. In this study, the typical grain boundary features of the four deformed ceramics are shown in Figure 6. Unlike the undoped ZTA ceramic with a clean grain boundary (Figure 6a), grain boundary liquid phases with different features are found in the deformed TiO_2_-doped ZTA ceramics, as shown in Figure 6b–d. For the 1 wt.% TiO_2_-doped ZTA ceramic, several thin liquid grain boundary phases (thickness is approximate 1–2 nm) can be observed at A-Z (meaning Al_2_O_3_-ZrO_2_ interfaces), A-A, and Z-Z interfaces. Notably, not all grain boundaries are covered by the thin liquid phases. Some grain boundaries are still clean. For 4 wt.% and 8 wt.%TiO_2_-doped ZTA ceramics, besides the thin grain boundary liquid phase, lots of thick grain boundary liquid phases (the thickness is 50–100 nm) (Figure 6c,d) were also observed at grain boundaries and triple junctions. Therefore, with the increase of TiO_2_ content, the grain boundary diffusion should be improved significantly due to the changing of the grain boundary feature, which is according to the decrease of the apparent activation energy and increase of the strain rate at a given temperature and stress during deformation.

## 4. Conclusions

In this study, the compressive deformation of the TiO_2_-doped ZTA ceramics at different temperatures was conducted. In the range of 1300–1400 °C, the values of stress exponents are 3.06–3.18 for 0T, 2.87–2.92 for 1T, 2.35–2.44 for 4T, and 2.03–2.16 for 8T, respectively. By doping TiO_2_, the apparent deformation activation energy of the ZTA ceramics decreased gradually from 749 kJ/mol to 605 kJ/mol, and the grain boundary feature was changed from clean to glassy. Based on the deformation behavior and microstructure evolution, the lower apparent activation energy and higher strain rate in TiO_2_-doped ZTA ceramics are intensively related to the grain boundary feature.
